# Insights into the photovoltaic properties of indium sulfide as an electron transport material in perovskite solar cells

**DOI:** 10.1038/s41598-023-36427-3

**Published:** 2023-06-05

**Authors:** Davoud Dastan, Mustafa K. A. Mohammed, Ali K. Al-Mousoi, Anjan Kumar, Sinan Q. Salih, P. S. JosephNg, Duha S. Ahmed, Rahul Pandey, Zaher Mundher Yaseen, M. Khalid Hossain

**Affiliations:** 1 Department of Materials Science and Engineering, Cornell University, Ithaca, NY 14850 USA; 2 University of Warith Al-Anbiyaa, 56001 Karbala, Iraq; 3 Electrical Engineering Department, College of Engineering, Al-Iraqia University, Baghdad, 10011 Iraq; 4 Solar Lab, GLA University, Mathura, 281406 India; 5 Technical College of Engineering, Al-Bayan University, Baghdad, 10011 Iraq; 6 Faculty of Data Science & Information Technology, INTI International University, Persiaran Perdana BBN, 71800 Nilai, Negeri Sembilan Malaysia; 7 Applied Sciences Department, University of Technology-Iraq, Baghdad, 10011 Iraq; 8 VLSI Centre of Excellence, Chitkara University Institute of Engineering and Technology, Chitkara University, Rajpura, Punjab 140417 India; 9 Civil and Environmental Engineering Department, King Fahd University of Petroleum & Minerals, Dhahran, 31261 Saudi Arabia; 10 Interdisciplinary Research Center for Membranes and Water Security, King Fahd University of Petroleum & Minerals, Dhahran, 31261 Saudi Arabia; 11 Institute of Electronics. Atomic Energy Research Establishment, Bangladesh Atomic Energy Commission, Dhaka, 1349 Bangladesh

**Keywords:** Energy science and technology, Materials science, Materials for devices

## Abstract

According to recent reports, planar structure-based organometallic perovskite solar cells (OPSCs) have achieved remarkable power conversion efficiency (PCE), making them very competitive with the more traditional silicon photovoltaics. A complete understanding of OPSCs and their individual parts is still necessary for further enhancement in PCE. In this work, indium sulfide (In_2_S_3_)-based planar heterojunction OPSCs were proposed and simulated with the SCAPS (a Solar Cell Capacitance Simulator)-1D programme. Initially, OPSC performance was calibrated with the experimentally fabricated architecture (FTO/In_2_S_3_/MAPbI_3_/Spiro-OMeTAD/Au) to evaluate the optimum parameters of each layer. The numerical calculations showed a significant dependence of PCE on the thickness and defect density of the MAPbI_3_ absorber material. The results showed that as the perovskite layer thickness increased, the PCE improved gradually but subsequently reached a maximum at thicknesses greater than 500 nm. Moreover, parameters involving the series resistance as well as the shunt resistance were recognized to affect the performance of the OPSC. Most importantly, a champion PCE of over 20% was yielded under the optimistic simulation conditions. Overall, the OPSC performed better between 20 and 30 °C, and its efficiency rapidly decreases above that temperature.

## Introduction

The scientific community has shown a great deal of interest in researching perovskite solar cells (OPSCs), which are mainly comprised of organic–inorganic metal halide compounds and are used to produce high-efficiency and inexpensive photovoltaic (PV) technologies^1–3^. These semiconductors have a number of important characteristics, including high charge carrier mobility, long carrier diffusion length, adjustable bandgaps, and a high absorption coefficient^4–7^. Due to such exceptional properties, photoconversion efficiency (PCE) values spiked substantially, from 3.8% in 2009 to over 25% in 2021^8–11^. In order, an OPSC has a front electrode, an electron-transport material (ETM), a light harvesting layer, a hole-transport material (HTM), and a back electrode. The harvester material of an OPSC generates charge carriers when exposed to sunlight^12–15^. These photocarriers are delivered to the appropriate electrodes by ETMs and HTMs. The relevance of charge transport materials is crucial to the entire PV performance of OPSCs, in addition to the perovskite layer's role. For instance, titanium dioxide (TiO_2_), a common ETM, is not suitable for fabricating large devices since it demands an operating temperature of more than 400 °C. The use of TiO_2_ in high-efficiency OPSCs is further limited by the material's poor electron mobility (µ_e_) and UV instability^16–18^. This highlights the need to look for a candidate ETM layer with appropriate properties, such as high µ_e_, good electrical conductivity (σ), and low-temperature manufacturing.

Compact ETM-based planar PSCs have a simplified layout and are easier to fabricate. TiO_2_ and ZnO have been widely used as ETMs for planar n-i-p OPSCs^19–23^. Nevertheless, planar OPSCs based on compacted TiO_2_ and ZnO often exhibit low stability because of the materials' limited carrier mobility, offset energy level alignment with perovskites, and defect traps at the surface^24–28^. As a result, it is important to provide cutting-edge ETM components for OPSCs. Indium sulfide (In_2_S_3_) is an n-type semiconductor with excellent carrier mobility, nontoxicity, an adequate bandgap, adjustable electrical properties, and good thermal durability^29,30^, all of which are ideal for utilization as an ETM in solar cells^31,32^. By adjusting the period of chemical bath deposition to 2 h, Hou et al. were able to construct an In_2_S_3_ nanoflakes array as ETMs for CH_3_NH_3_PbI_3_ OPSCs, achieving a performance of 18.22%. However, the long-term stability of In_2_S_3_-OPSC was not examined in this work^30^. One year later, Xu et al. prepared In_2_S_3_ sheets as ETMs for CH_3_NH_3_PbI_3_ devices using a solvent-thermal approach for 2 h and achieved an efficiency of 18.83%^33^. Subsequently, Yang et al. made further efforts to use In_2_S_3_ film and developed a spray-assisted deposition technique as an ETM for semitransparent CsPbIBr_2_ OPSCs. The optimized devices obtained a performance of 5.59% with improved ambient stability^34^. Meanwhile, as far as we can tell, no theoretical studies relevant to adopting In_2_S_3_ as the ETM in perovskite solar cells have been reported.

In this work, the first ever conventional n-i-p planar architecture of OPSCs using In_2_S_3_ as the electron transport material has been simulated and optimized. To verify our data, we recreated the findings of an experimentally published 18.83% robust and stable single-cation OPSC (FTO/In_2_S_3_/MAPbI_3_/Spiro-OMeTAD/Au)^33^. To improve the efficiency of the control OPSC, the thickness variation of the perovskite (*t*_p_) was further investigated. Along with thickness variation, the effects of defect density (*N*_T_), series resistance (*R*_s_), shunt resistance (*R*_sh_), and operating temperature on OPSC performance were studied. Our research can offer some key advice for OPSC design and optimization based on theoretical principles.

## Method and simulation

The numerical modeling of the devices enables us to understand the solar cell dynamics without the need for actual manufacturing. It also provides a high-level outline of the device's functionality. The one-dimensional SCAPS (version 3.3.07) was used in this simulation study. In 2000, researchers at the University of Gent in Belgium created this open-source program, which can be downloaded at any time^35^. The SCAPS software assists in the modeling of planar and graded PV structures up to seven components, with the additional functionality of calculating the band alignment graph, current–voltage (*J–V*) behavior, quantum efficiency (QE), recombination and generation currents, and other essential PV characteristics. SCAPS-1D relies primarily on the well-established Poisson's formula and the continuity laws for electrons and holes to perform its calculations^36–39^. SCAPS is very powerful software for performing solar cell and a description of the programme, and the algorithms it uses, is found in the literature^40,41^ and in its user manual^42^.

1 $$\mathrm{Poisson \; equation}{:}\; -\frac{\partial }{\partial x}\left(\varepsilon \left(x\right)\frac{\partial V}{\partial x}\right)=q[p\left(x\right)n\left(x\right)+{N}_{D}^{+}\left(x\right)-{N}_{A}^{-}\left(x\right)+{p}_{t}\left(x\right)-{n}_{t}\left(x\right)]$$

2 $$\mathrm{Continuity \; equation \; for\; the \;hole}{:}\; \frac{\partial p }{\partial t} =\frac{1}{q} \frac{\partial {J}_{p}}{\partial x}+{G}_{p}- {R}_{p}$$

3 $$\mathrm{Continuity \; equation \; for \;the \;electron}{:}\; \frac{\partial n }{\partial t} =\frac{1}{q} \frac{\partial {J}_{n}}{\partial x}+{G}_{n}- {R}_{n}$$

where *q* is the charge, *V* is the potential, *p(x)* is the free hole concentration, *n(x)* is the free electron concentration,* ε* is the dielectric permittivity, ${N}_{D}^{+}\left(x\right)$ is the donor density, ${N}_{A}^{-}\left(x\right)$ is the acceptor density, *p*_*t*_*(x)* is the hole trap concentration, *n*_*t*_*(x)* is the trap concentration of an electron, *J*_*n*_ is the current density of an electron, *J*_*p*_ is the current density of a hole, *G*_*n*_ is the electron generation rate, *G*_*p*_ is the holes generation rate, *R*_*n*_ is the recombination rate of electrons, *R*_*p*_ is the recombination rate of holes.

**Figure 1:**
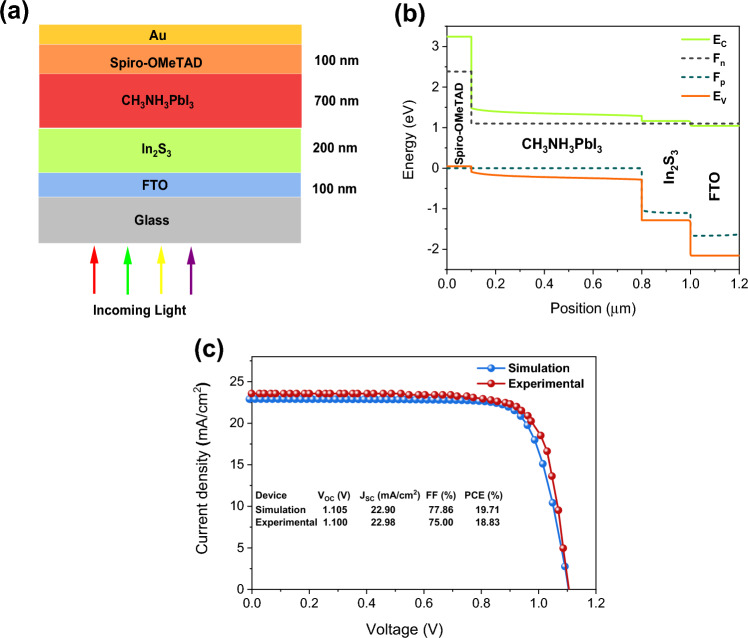
(**a**) Solar cell structure utilized in simulation. (**b**) Band alignment diagram of the proposed OPSC. (**c**) A representation comparison between experimental and modeled parameters of our control OPSC^33^.

Here, we simulated a typical n-i-p PV architecture with CH3NH3PbI3 perovskite as the photoactive film, compact In_2_S_3_ as the ETM, and Spiro-OMeTAD organic film as the HTM, with fluorine-containing SnO_2_ (FTO) and gold (Au) as the front and back electrodes, respectively. In Fig. 1a, we have a graphical diagram of the FTO/In_2_S_3_/CH_3_NH_3_PbI_3_/Spiro-OMeTAD/Au device assembly. Tables 1 and 2 summarize the fundamental device parameters of several materials utilized in this analysis that were acquired from the theoretical and experimental literature. The work functions for the front and back electrodes were 4.4 eV and 5.2 eV, respectively. The SCAPS software calculated the absorption spectrum of each layer based on the optical merits of the materials and the geometry of the device.

**Table 1:** The input parameters of simulated perovskite solar cells.

Device layer properties	Device layers				
	Unit	In_2_S_3_	IGZO	MAPbI_3_	Spiro-OMeTAD
Thickness	nm	200	200	700 (varied)	100
Energy gap	eV	2.45	3.05	1.57	3.2
Electron affinity energy	eV	3.98	4.16	3.86	2.1
µ_e_	cm^2^/V s	400	15.0	8	2 × 10^4^
µ_h_	cm^2^/V s	210	0.1	4	2 × 10^4^
Concentration of the shallow acceptor (*N*_A_)	1/cm^3^	0	0.0	1 × 10^19^ (varied)	1 × 10^20^
Concentration of the shallow donor (*N*_D_)	1/cm^3^	1.47 × 10^18^	1.0 × 10^18^	1 × 10^19^	0
CB effective density of states (*N*_C_)	1/cm^3^	1.8 × 10^19^	5.8 × 10^18^	1 × 10^18^	2.8 × 10^18^
VB effective density of states (*N*_V_)	1/cm^3^	4 × 10^13^	5.8 × 10^18^	1 × 10^18^	1.8 × 10^19^
Dielectric permittivity	–	6.5	10.0	28	3
Defect type	–	Neutral	Neutral	Neutral	Neutral
Capture cross section of electrons	cm^2^	1 × 10^–15^	1 × 10^–15^	1 × 10^–15^	1 × 10^–15^
Capture cross section of holes	cm^2^	1 × 10^–15^	1 × 10^–15^	1 × 10^–15^	1 × 10^–15^
*N*_T_	1/cm^3^	1 × 10^16^ (varied)	1.0 × 10^15^	2.45 × 10^15^ (varied)	1 × 10^14^
Energetic distribution	–	Single	Single	Single 0.6 eV above E_v_	Single 0.6 eV above E_v_
References		^43^	^44^	^45^	^46^

**Table 2:** Interface parameters of FTO/ In_2_S_3_/MAPbI_3_/Spiro-OMeTAD/Au OPSCs.

Parameters/interfaces	In_2_S_3_/MAPbI_3_	MAPbI_3_/spiro-OMeTAD
Defect type	Neutral	Neutral
Total defect density (cm^−2^)	1 × 10^10^	1 × 10^10^
Capture cross section electrons (cm^2^)	1 × 10^10^	1 × 10^−18^
Capture cross section holes (cm^2^)	1 × 10^−19^	1 × 10^−18^
Energetic distribution	Single	single
Reference for defect energy level E_t_	Above the highest E_v_	Above the highest E_v_
Energy with respect to reference (eV)	0.600	0.600
Total density (1/cm^2^)	1 × 10^10^	1 × 10^10^

The defects were used 0.6 eV above the valence band with a particular energy of 0.1 eV, taking into account the Gaussian energy distribution and the capture cross-section of carriers of 10^–15^ cm^2^. The radiative recombination coefficient for perovskite was 2.3 × 10^–9^ cm^3^/s, which was taken into consideration. The modeling analysis added imperfections at the HTM/perovskite and perovskite/ETM interfaces about 10^10^ cm^−2^. The conventional AM 1.5 G spectrum and a temperature of 300 K were used for the computations.

Figure 1b displays the band structure diagram for the suggested n-i-p OPSC layout. At the conduction band interface of In_2_S_3_ and CH_3_NH_3_PbI_3_, a potential barrier of 0.12 eV exists, which is a beneficial barrier for the better transport of electrons from perovskite to ETM, whereas at the junction of the valence band of perovskite and HTM, holes have to contend with a large barrier of 0.13 eV. The *J–V* plot of the suggested cell architecture was analyzed after the appropriate layer parameters and operational conditions were determined (as covered in this section). Figure 1c displays the calculated J–V graph and its initial output parameters. We attained a power conversion efficiency (PCE) of 19.71%, which is close to the PCE of 18.83% that has been published experimentally^33^. A slight mismatch between the experimental and computed results is that in the present research, the FTO and Au layers were utilized as front and back electrodes, where the thickness of front and back contacts cannot be changed. In the experimental research, however, they were employed as layers with appropriate thicknesses.

### Ethics approval and consent to participate

This article does not contain any studies with human participants or animals performed by the authors. We comply with the ethical standards. We provide our consent to take part.

## Results and discussion

**Figure 2:**
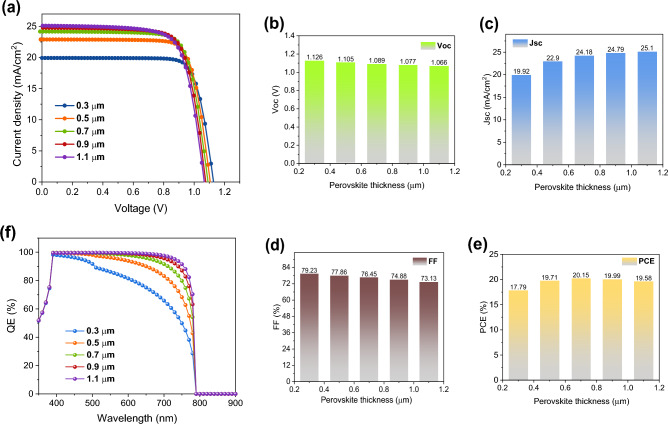
(**a**) *J–V* characteristics of the OPSCs with different MAPbI_3_ thicknesses. Variations of OPSC performance parameters with various thicknesses of perovskite: (**b**) *V*_OC_, (**c**) *J*_SC_, (**d**) FF, and (**e**) PCE. (**f**) QE of devices with various MAPbI_3_ thicknesses.

Increasing the device's efficiency is highly dependent on the thickness of the absorber layer. Nevertheless, using a very thick photoactive layer leads to a low charge carrier extraction rate and considerable losses owing to charge recombination; finding the right equilibrium between these two variations is crucial. Therefore, optimizing the light-absorbing thickness becomes essential for determining photocarrier production and spectrum response in photovoltaics^47^. The obtained *J–V* graphs are shown in Fig. 2a, with perovskite thickness variations ranging from 0.3 to 1.1 µm, whereas Fig. 2b–e show the variations in the *J*_SC_, *V*_OC_, FF, and PCE parameters. According to Fig. 2, an increment in the perovskite thickness causes a rise in the *J*_SC_ and a reduction in the *V*_OC_. The trend of rising *J*_SC_ values is a result of increased photocarrier production. Thin perovskite film results in lower long-wavelength photon absorption rates, which results in less photocarrier formation and worse *J*_SC_ values^48^. Furthermore, the poor recombination due to the thin perovskite creates a high *V*_OC_. Increasing the absorber perovskite's thickness also boosts the layer's ability to absorb light with longer wavelengths. As a result, more charge carriers are produced, which leads to a rise in the value of the *J*_SC_^49^. However, with higher absorbance, the recombination rate of photocarriers also increases since photocarriers have to cover a longer distance before approaching the corresponding electrodes. The increase in perovskite thickness raises the *R*_s_, which causes a decrease in FF. The improvement in efficiency is attributable to the steady rise in *J*_SC_. Our calculations suggest that the ideal value for the perovskite thickness should be 0.7 µm for the highest performance of MAPbI_3_-based single-cation OPSC. Therefore, optimizing the thickness of the perovskite layer is crucial for achieving the highest efficiency in a perovskite solar cell. By carefully balancing the absorption of light and the extraction of charge carriers, an optimal thickness can be found that maximizes the photocurrent and minimizes recombination, leading to the best performance of the device.

**Figure 3:**
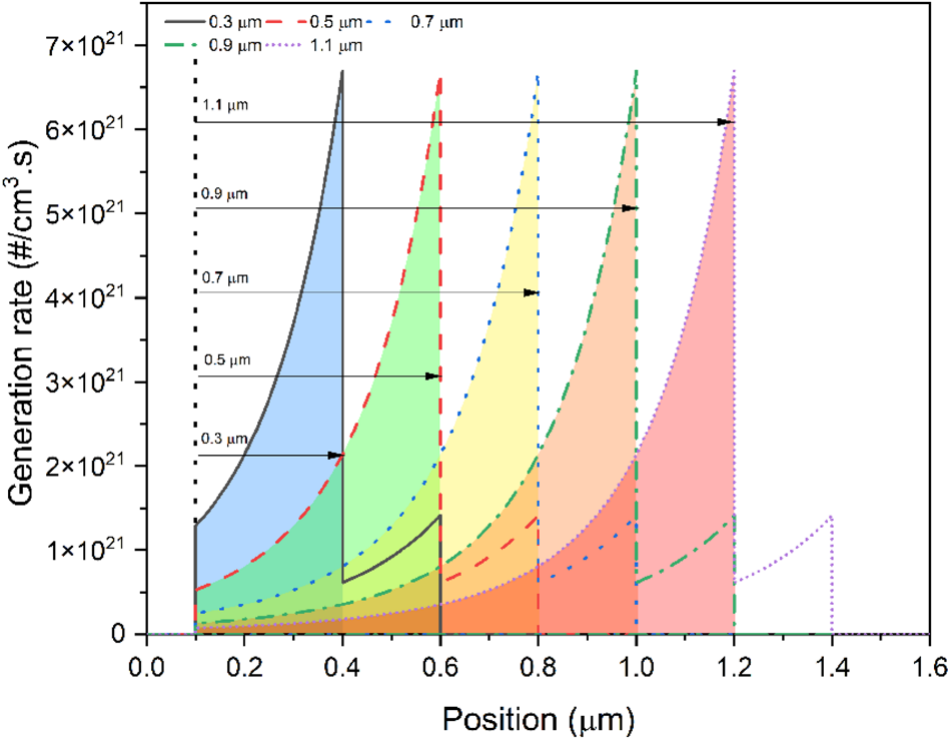
Generation rate inside the device with at different thicknesses of the absorber layer.

Figure 2f illustrates the external QE (EQE) of devices with varying MAPbI_3_ film thicknesses. The EQE of the device was clearly improved when the MAPbI_3_ light harvester thickness was less than 0.7 µm, which indicates that the improvement in absorption was high. Nevertheless, the EQE of the device rose less when the thickness of MAPbI_3_ was more than 0.7 µm, indicating that the rise in absorption was less significant. As the MAPbI_3_ film thickness increased, it was better able to absorb light of longer wavelengths^50^. The profile of carrier generation rate is also obtained and reported in Fig. 3 to validate the higher penetration of generation rate in the absorber layer at higher thicknesses.

**Figure 4:**
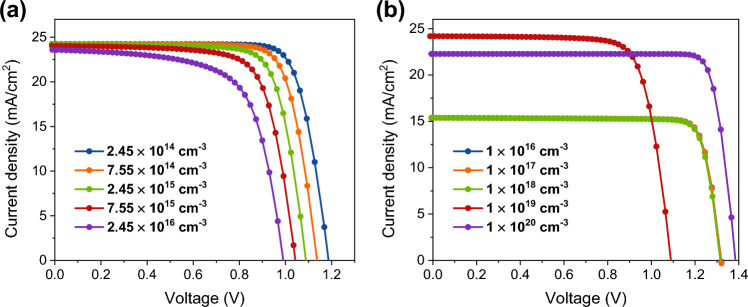
*J–V* plots of the OPSCs obtained with varying (**a**) total defect density and (**b**) concentration of the shallow acceptor in CH_3_NH_3_PbI_3_.

**Figure 5:**
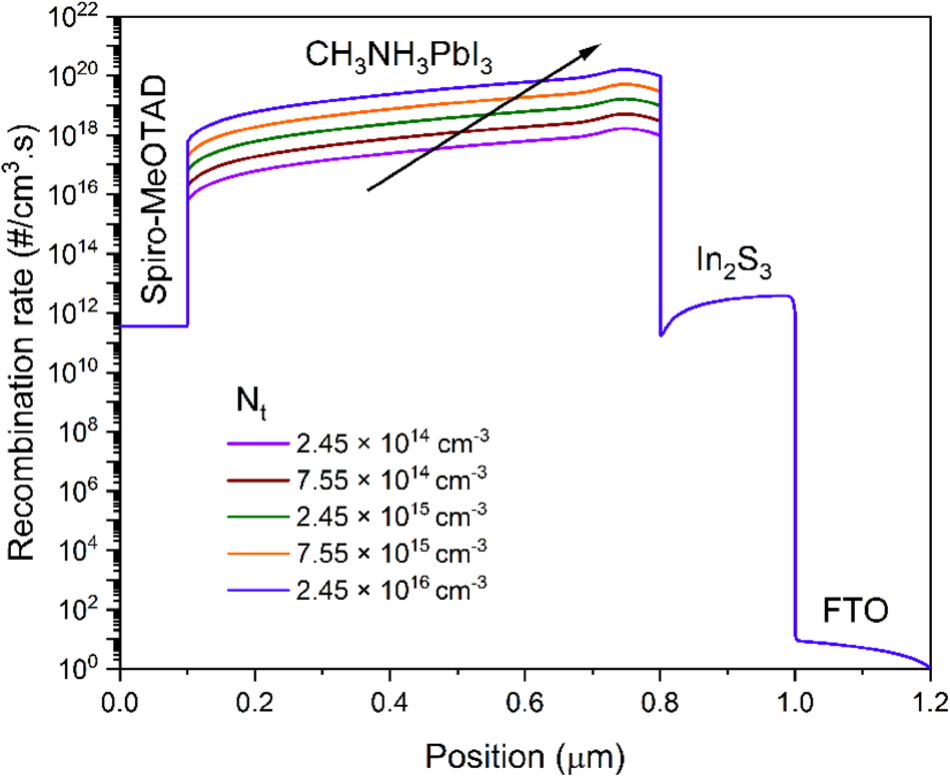
Recombination rate profile at different defect density in absorber layer.

The number of defects in the photoactive MAPbI_3_ has a significant impact on the output quality of perovskite solar cells. The *V*_OC_ of the device may be optimized by controlling the generation-recombination rate of the photocarriers inside the perovskite. Shockley–Read–Hall (SRH) recombination may provide a more adequate explanation for the correlation between *N*_T_ and OPSC performance^37,49^. The perovskite defect density in this analysis ranges from 2.45 × 10^14^ to 2.45 × 10^16^ cm^−3^, and its impact on how well our computed work performs is investigated. Figure 4a displays *J–V* graphs that have been plotted with varying *N*_T_ values. Results show that a minor decrease in *J*_SC_—from 24.241 to 23.582 mA/cm^2^ and a major reduction in *V*_OC_—from 1.188 to 0.991 V—are found when the *N*_T_ is increased from 2.45 × 10^14^ to 2.45 × 10^16^ cm^−3^ (Table 3). Since FF is dependent on *V*_OC_, there is a significant decrease in FF values (from 79.163 to 66.498%). The efficiency was dramatically reduced from 22.79 to 15.55% because of these decreases in *J*_SC_, *V*_OC_, and FF values. This suggests that a rise in the *N*_T_ values leads to a greater number of imperfections, which in turn raises the recombination process, as shown in Fig. 5. According to the experimentally stated value, we chose the *N*_T_ for the perovskite here to be 2.45 × 10^15^ cm^−3^, which accounts for carrier diffusion lengths (*L*_p_) of photocarriers of about 0.65 µm^33^.

**Table 3:** PV device parameters of OPSCs with varying total defect density in CH_3_NH_3_PbI_3_.

*N*_T_ (cm^−3^)	*L*_p_ (µm)	*V*_OC_ (V)	*J*_SC_ (mA/cm^2^)	FF (%)	PCE (%)
2.45 × 10^14^	2.1	1.188	24.241	79.163	22.79
7.55 × 10^14^	1.2	1.138	24.227	78.347	21.60
2.45 × 10^15^	0.65	1.089	24.180	76.456	20.15
7.55 × 10^15^	0.37	1.043	24.039	73.047	18.32
2.45 × 10^16^	0.21	0.991	23.582	66.498	15.55

The efficiency of OPSC is significantly affected by the amount of doping used. Doping can be categorized as either n-type or p-type, depending on the dopants used. Thus, improving OPSC efficiency relies on setting the appropriate value of *N*_A_. Doping concentration levels can be adjusted experimentally in many different ways^51^. Doping concentrations and defect density values, for example, can be experimentally modified by adding different dopants or adjusting their concentrations in the perovskite material. Experimentally changing doping ratios and minimizing defects may also be accomplished by adjusting the relative amounts of cesium (Cs), methylammonium iodide (MAI), formamidinium iodide (FAI), and lead iodide (PbI_2_)^52^.

Furthermore, the *N*_A_ of the perovskite was adjusted from 10^16^ to 10^20^ cm^−3^, and the results are shown in Fig. 4b to help understand the impact of doping on the OPSC performance. According to our findings, the *J–V* characteristics are unchanged at low *N*_A_ levels. Nevertheless, the inherent built-in electric field (*V*_bi_) rises when *N*_A_ surpasses 10^18^ cm^−3^. The performance of the cell is enhanced by an increase in *V*_bi_ because it leads to improved separation of photocarriers. *J*_SC_ was shown to decrease with increasing *N*_A_ levels (Table 4). Auger recombination might explain a decline in *J*_SC_ value with rising *N*_A_. Auger recombination rises with increasing doping ratios, which lowers device efficiency^53,54^. Here, a further decline in *J*_SC_ was shown if the *N*_A_ was raised above 10^19^ cm^−3^. As a result, we decided to set the highest value for N_A_ in the current simulation at 10^19^ cm^−3^.

**Table 4:** PV device parameters of OPSCs with varying concentration of the shallow acceptor in CH_3_NH_3_PbI_3_.

*N*_A_ (cm^−3^)	*V*_OC_ (V)	*J*_SC_ (mA/cm^2^)	FF (%)	PCE (%)
1 × 10^16^	1.319	15.382	85.548	17.36
1 × 10^17^	1.319	15.382	85.523	17.35
1 × 10^18^	1.316	15.381	85.236	17.26
1 × 10^19^	1.090	24.180	76.469	20.16
1 × 10^20^	1.386	22.270	86.143	26.60

**Figure 6:**
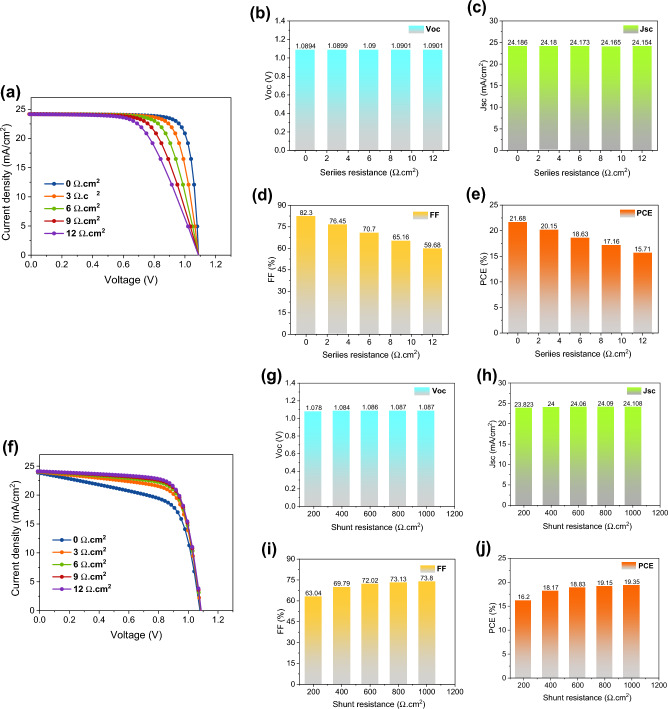
(**a**) *J–V* characteristics of the OPSCs with different series resistances. Variations of OPSC performance parameters with various series resistances: (**b**) *V*_OC_, (**c**) *J*_SC_, (**d**) FF, and (**e**) PCE. (**f**) *J–V* characteristics of the OPSCs with different shunt resistances with constant series resistance of 3 Ω cm^2^. Variations of OPSC performance parameters with various shunt resistances: (**g**) *V*_OC_, (**h**) *J*_SC_, (**i**) FF, and (**j**) PCE.

The series resistance (*R*_s_) has a major effect on the operation of the OPSC, particularly the FF and short circuit current (*I*_SC_). When the resistance of a series circuit rises, FF drops. Therefore, for higher levels of *R*_s_, the *I*_SC_ begins to decrease as well. Hence, a device's efficiency suffers when *R*_s_ is quite high^55^. This led researchers to examine how the PCE and FF of perovskite photoactive material changed with variations in *R*_s_. We evaluate the performance of the OPSC while changing the *R*_s_ from 0 to 12 Ω cm^2^ to examine the impact of *R*_s_ on OPSC performance. The *J–V* profiles for various resistances are depicted in Fig. 6a. Our research shows that the photovoltaic has superior performance and a higher FF at lower *R*_s_ (Fig. 6b–e). The efficiency of the devices deteriorates rapidly as the *R*_s_ rises. These findings are consistent with those reported in other studies^36,56^.

Shunt resistance (*R*_sh_) is caused by the several pathways for charge recombination in the OPSC^57^. We simulate the device's operation, changing the *R*_sh_ from 0 to 1000 Ω cm^2^, to examine the impact of *R*_sh_ on OPSC performance. Changing *R*_sh_ affects several different device characteristics, as seen in Fig. 6f,j. The performance of OPSC is found to improve as *R*_sh_ rises. PCE = 19.15% and FF = 73.13% at 800 Ω cm^2^, and at 1000 Ω cm^2^ we obtain PCE = 19.35% and FF = 73.8%, respectively. Therefore, we determine that an *R*_sh_ of 800 Ω cm^2^ is optimal.

**Figure 7:**
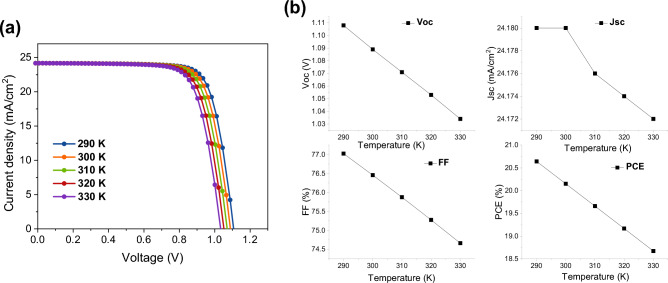
(**a**) Effect of operational temperature with respect to (**a**) *J–V* curves and (**b**) PV parameters (*J*_SC_, *V*_OC_, FF, and PCE).

Figure 7a illustrates how altering the ambient temperature from 17 to 57 °C has an impact on the *J–V* plots of the OPSC device. It turns out that both *V*_OC_ and FF suffer when the temperature goes up. However, there are not any noticeable changes at *J*_SC_. Efficiency gradually drops because both *V*_OC_ and FF are impacted by rising temperatures. This investigation demonstrates that OPSC in an ambient environment gives better efficiency, which is over 25%; however, as the temperature rises, this efficiency gradually declines, as shown in Fig. 7b. An increase in temperature increases the recombination and reverse saturation currents, which further reduce the *V*_OC_ and device performance. In addition, when the device is running at a higher temperature, the bandgap gets smaller, which may lead to more exciton recombination and less efficiency^58^. This observation may be extremely important when choosing OPSC in tropical areas.

**Figure 8:**
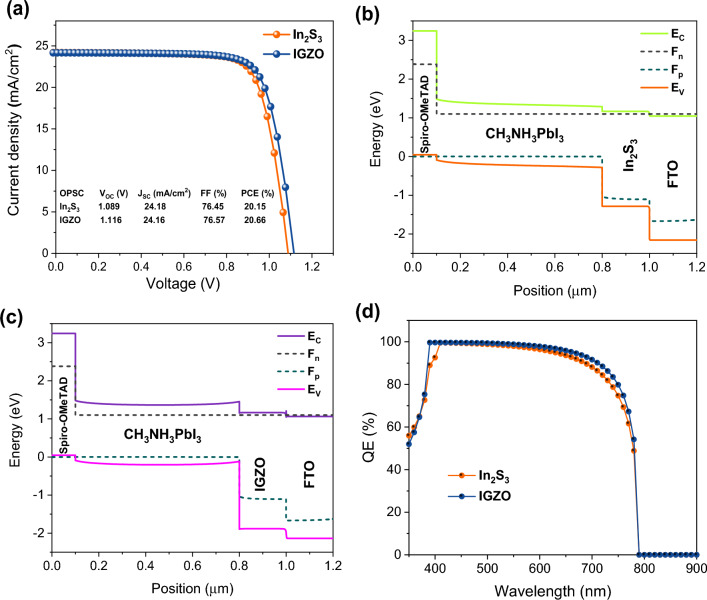
(**a**) *J–V* curves of perovskite solar cells with different ETMs, including In_2_S_3_ and IGZO films. Band offset behaviour of the proposed OPSC based on (**b**) In_2_S_3_ and (**c**) IGZO. The diagram was calculated using optimized thicknesses of ETMs (200 nm) and MAPbI_3_ layer (700 nm). (**d**) EQE of OPSCs with various ETMs at a thickness of 200 nm.

Finally, the performance of the optimized OPSC was compared to that of an OPSC made of indium gallium zinc oxide (IGZO) as an ETM layer (see Fig. 8a). Recently, IGZO has been used as an ETM; it offers great promise because of its high *µ*_e_, environmental stability, low processing temperatures, and comparable electron affinity to perovskite^37,44,59^. As we can see in inset table of Fig. 8, In_2_S_3_-based device showed comparable photovoltaic parameters to the IGZO-based device. The findings from this study are expected to facilitate the manufacturing of high-efficiency perovskite solar cells in the near future. The energy level layout is constructed by incorporating an ETM, a MAPbI_3_ absorbing layer, and Spiro-OMeTAD as HTM. This arrangement affects the valence/conduction band offset, which refers to the variation in the valence band between the HTM and the perovskite, as well as the conduction band between the ETM and the perovskite. The energy level offset at the ETM/MAPbI_3_ and the MAPbI_3_/HTM interfaces greatly affects the solar cell’s performance^36^. Figure 8b,c shows that quasi-Fermi levels *F*_n_ and *F*_p_ coexist with *E*_C_ and *E*_V_ in the OPSCs based on In_2_S_3_ and IGZO layers. As shown, the In_2_S_3_- and IGZO-based structures showed a small conduction band offset (CBO) of 0.121 eV and 0.294 eV at ETM/MAPbI_3_ interface, indicating that In_2_S_3_ ETM provides better interface for electron transportation. However, IGZO film showed larger valence band offset at ETM/MAPbI_3_ interface, which is significant for blocking the backflow of holes and suppressing the recombination rate in the OPSC.

Finally, we estimated The EQE spectra of In_2_S_3_ and IGZO ETM-based OPSCs, as shown in Fig. 8d. The EQE could vary depending on the specific features of the semiconductors and the design of the cell. IGZO ETM-based OPSC has been proven to demonstrate relatively higher QE spectrum across the visible spectrum than In_2_S_3_ ETM-based OPSC. This is because IGZO has a wide bandgap, which allows it to absorb a minimal amount of visible light while still effectively extracting electrons from the MAPbI_3_ film. In general, it can be concluded that the utilization of both In2S3 and IGZO ETMs can effectively enhance the EQE of OPSCs. However, the selection of a suitable ETM is dependent upon the specific needs of the device and the preferred wavelength range for optimal performance.

We have provided insights into the relationship between the device's performance and the defects' density, which could be useful for optimizing the fabrication process and improving the device's performance. One possible approach to address this issue is to optimize the growth conditions during the fabrication process to minimize the defect density. For example, by carefully controlling the temperature, pressure, and some other important parameters of spin coating method during the growth process, it is possible to reduce the number of defects in the device. Interface passivation and anion/cation engineering can also be done to reduce the defect density. Additionally, post-growth processing techniques such as annealing could also reduce the density of defects in the material. In summary, we agree that the feasibility of tuning the property of the device at the fabrication or industrial level is an important consideration.

## Conclusions

For the first time, the SCAPS-1D model has explored the potential of In_2_S_3_ as an alternate ETM film in OPSCs in an effort to increase PV stability, boost efficiency, and reduce hysteresis behavior. Problems with imperfections and high temperatures are fundamental to the simulation analysis. Theoretically, In_2_S_3_ can substitute TiO_2_ as ETL in OPSC, and the results showed that defect states have a significant impact on OPSC efficiency at defect densities higher than 2.45 × 10^15^ cm^−3^. Finally, OPSC works best between 20 and 30 °C. The optimized design with an efficiency of 20.15% (*V*_OC_ = 1.089 V, *J*_SC_ = 24.18 mA/cm^2^, and FF = 76.45%) sheds light on the possibility of In_2_S_3_ as a suitable ETL. This study paves the way towards practical implementation of indium sulfide as the potential ETL for MAPbI_3_ perovskite solar cells.

## Data Availability

The datasets used and/or analysed during the current study available from the corresponding author on reasonable request.
